# *GastroAlert*: a multimodal epigenetic blood test for early detection of gastric cancer using cell-free DNA

**DOI:** 10.1016/j.mmr.2026.100066

**Published:** 2026-08-25

**Authors:** Yong-Jie Xu, Jian-Feng Xu, Chang-Fa Xia, Yu-Jie Wu, Dian-Ge Li, Tie-Yan Liu, Huai-Ping Cui, Jin Zhang, Liang Shang, Wan-Qing Chen

**Affiliations:** a Office of Cancer Screening, National Cancer Center/National Clinical Research Center for Cancer/Cancer Hospital, Chinese Academy of Medical Sciences and Peking Union Medical College, Beijing 100021, China; b Beijing Kangshu Medical Technology Co., Ltd., Beijing 100176, China; c Department of Gastroenterological Surgery, Shandong Provincial Hospital of Shandong First Medical University, Jinan 250021, China; d Cixian Institute for Cancer Prevention and Control, Cixian Cancer Hospital, Handan 056500, Hebei, China; e Office of Cancer Registry, National Cancer Center/National Clinical Research Center for Cancer/Cancer Hospital, Chinese Academy of Medical Sciences and Peking Union Medical College, Beijing 100021, China

**Keywords:** Gastric cancer, Early detection, Cell-free DNA, Epigenetic biomarkers

## Abstract

**Background:**

Gastric cancer remains a leading cause of cancer-related mortality worldwide, frequently diagnosed at advanced stages due to the limitations of current diagnostic approaches. While cell-free DNA (cfDNA)-based epigenetic profiling has emerged as a promising avenue for early cancer detection, comprehensive investigations into the epigenetic landscape of cfDNA in gastric cancer remain scarce. To address this gap, we aimed to develop and validate a multimodal epigenetic blood test for the non-invasive detection of gastric cancer.

**Methods:**

We developed *GastroAlert*, a multimodal epigenetic blood test that integrates cfDNA methylation, nucleosome footprinting, and fragmentation features into a single diagnostic approach for gastric cancer detection. A custom-designed capture probe panel was utilized to target gastric cancer-associated differentially methylated CpG sites and functionally relevant genes. Targeted enzymatic methylation sequencing was then performed on a discovery dataset consisting of 136 participants, including 53 gastric cancer patients, 55 individuals with benign gastric conditions, and 28 healthy controls. Subsequently, the diagnostic performance of *GastroAlert* was rigorously validated in an independent external dataset of 149 participants, comprising 79 gastric cancer cases and 70 non-cancer controls.

**Results:**

In cross-validation of the discovery dataset, *GastroAlert* achieved an area under the receiver operating characteristic curve (AUC) of 0.950 [95% confidence interval (CI) 0.874–0.995], with observed AUCs for conventional protein biomarkers ranging from 0.507 to 0.687. Notably, the locked *GastroAlert* model exhibited robust and reproducible performance in the independent validation dataset, yielding an AUC of 0.965 (95% CI 0.940–0.989) for distinguishing gastric cancer patients from non-cancer controls. For the clinically critical subset of early-stage gastric cancer cases, the model still maintained high diagnostic efficacy with an AUC of 0.921 (95% CI 0.862–0.980) in the discovery dataset and 0.948 (95% CI 0.910–0.985) in the validation dataset. Among all 79 gastric cancer cases in the validation dataset, the model attained an overall sensitivity of 0.898 (95% CI 0.813–0.948) at a specificity of 0.900 in the validation dataset.

**Conclusions:**

The findings demonstrate that multimodal epigenetic analysis of cfDNA provides a robust, non-invasive strategy for early gastric cancer detection, with potential clinical utility to improve patient outcomes.

## 1 Background

Gastric cancer remains one of the leading causes of cancer-related mortality worldwide, with a poor prognosis largely due to late-stage diagnosis [1]. Early detection of gastric cancer is crucial for improving survival rates; however, current diagnostic methods, such as endoscopy and biopsy, are invasive and costly, suffering from poor compliance. This limits their effectiveness in early-stage detection [2]. Therefore, there is an urgent need for non-invasive biomarkers that can facilitate early diagnosis, enabling timely intervention and improving outcomes.

One promising biomarker for non-invasive cancer detection is cell-free DNA (cfDNA), a fragmented form of DNA that circulates in the bloodstream. cfDNA has shown great potential in reflecting the genetic and epigenetic changes occurring in tumors [3]. Specifically, methylation patterns in cfDNA have been widely studied as biomarkers in cancer detection, including gastric cancer [4], [5], [6]. Altered methylation, often specific to tumor DNA, reflects key epigenetic changes associated with cancer development [7]. These methylation alterations can provide valuable indicators for the early detection of gastric cancer.

In addition to methylation, other epigenetic features, such as DNA fragmentation patterns and nucleosome occupancy, are gaining recognition for their roles in cancer diagnostics. Tumor-derived cfDNA exhibits distinct fragmentation patterns, with specific sizes and distributions that can help differentiate cancerous from non-cancerous DNA [3], [8]. Furthermore, nucleosome footprinting, the alteration of nucleosome positioning on DNA, provides insights into the chromatin landscape of tumor cells, which is often disrupted in cancer [9], [10]. The integration of these multimodal epigenetic features therefore offers the potential for more comprehensive cfDNA analysis and is expected to improve cancer detection performance [11].

Conventional bisulfite sequencing, the current gold standard for methylation analysis, causes substantial DNA fragmentation and biased genome coverage, which not only compromises data quality but also precludes the simultaneous analysis of other cfDNA features. By contrast, targeted enzymatic methylation sequencing (EM‑seq) employs a mild enzymatic conversion that preserves DNA integrity and enables more uniform CpG coverage [12]. This non‑destructive nature allows for the simultaneous profiling of multiple cfDNA features from a single assay; however, its clinical utility for cancer detection remains to be fully established.

In this study, we employed deep targeted EM‑seq to simultaneously profile cfDNA methylation, nucleosome footprinting, and fragmentation features in gastric cancer patients within a single assay. We then aimed to integrate these multimodal features to develop a diagnostic model, termed *GastroAlert*, and evaluate its performance for gastric cancer detection, to establish a non‑invasive screening tool for this malignancy.

## 2 Methods

### 2.1 Participants enrollment

**Fig. 1:**
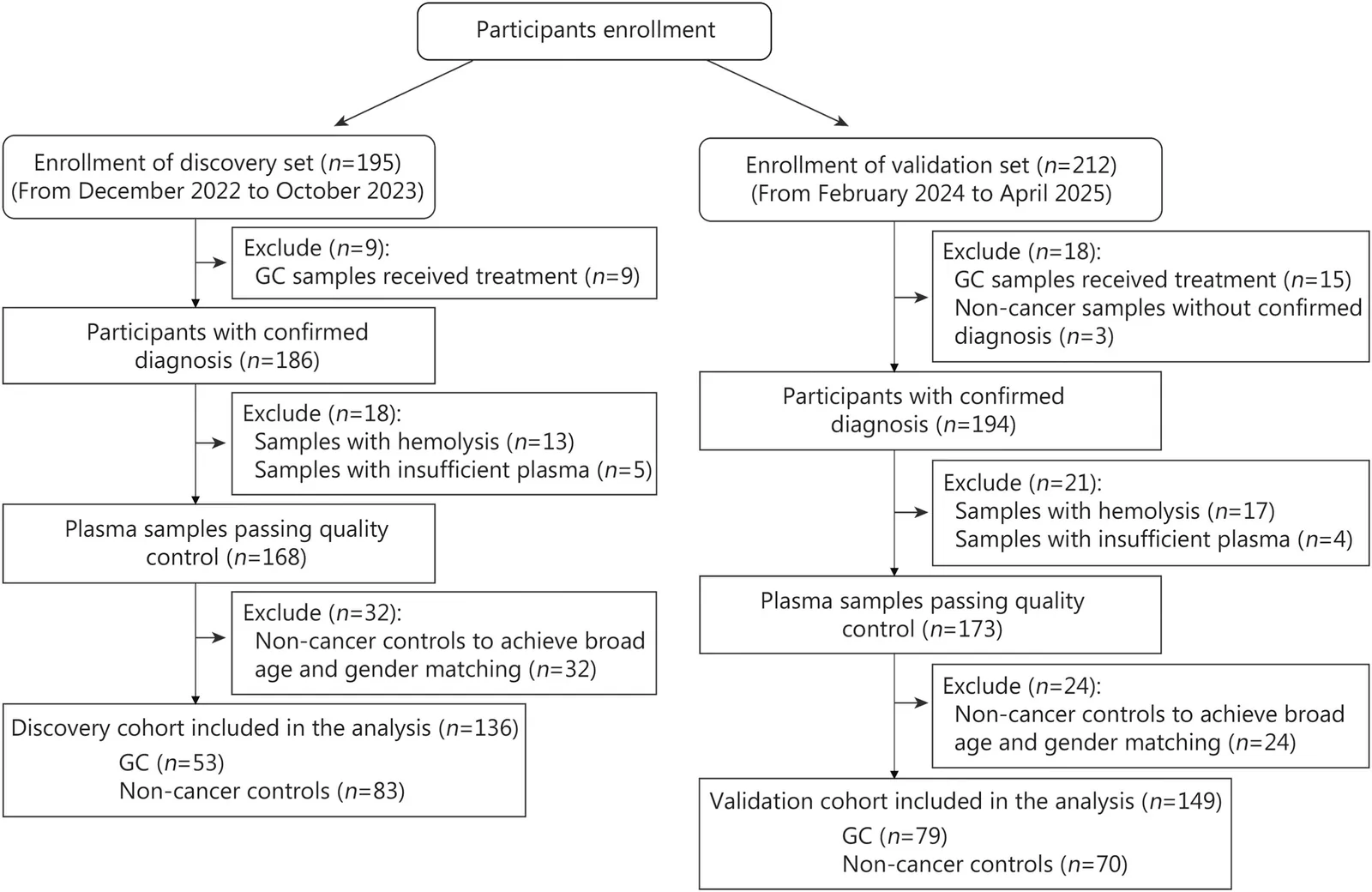
Flowchart of participant enrollment. GC. Gastric cancer.

To develop and evaluate a multimodal cfDNA epigenetic blood test for gastric cancer detection, we designed a case-control study comprising a discovery dataset and an independent external validation dataset (Fig. 1). Participants in the discovery dataset were enrolled between December 2022 and October 2023 at two clinical centers. Gastric cancer patients and patients with benign gastric diseases were recruited at the National Cancer Center/National Clinical Research Center for Cancer/Cancer Hospital, Chinese Academy of Medical Sciences and Peking Union Medical College, whereas healthy controls were enrolled at Shandong Provincial Hospital. Initially, a total of 195 participants were enrolled. After excluding individuals with prior anti‑cancer therapy, hemolyzed or insufficient plasma samples, and additional non‑cancer controls to balance age and sex distributions, a final dataset of 136 participants was included for cfDNA analysis, comprising 53 patients with gastric cancer, 55 patients with benign gastric diseases, and 28 healthy controls (Fig. 1**;** Table 1).

**Table 1:** Clinical characteristics of enrolled subjects for *GastroAlert* discovery dataset.

**Clinical indexes**	**Gastric cancer group (*****n*****=53)**	**Non-cancer group (*****n*****=83)**	***P*****-value**
Age (years, mean±SD)	60.66±10.27	56.97±7.53	0.034
Sex [*n* (%)]			0.258
Male	37 (69.81)	50 (60.24)	
Female	16 (30.19)	33 (39.76)	
Cancer stage [*n* (%)]			-
I	18 (33.96)	-	
II	19 (35.85)	-	
III	12 (22.64)	-	
IV	4 (7.55)	-	
Benign diseases [*n* (%)]			-
Chronic gastritis	-	32 (58.18)	
Gastric polyp	-	14 (25.45)	
LGIN & IM	-	9 (16.36)	
Tumor markers (mean±SD)			
CA19-9 (U/ml)	50.73±140.42	7.14±2.86	0.039
CA72-4 (U/ml)	6.19±9.23	4.46±6.68	1.000
CEA (ng/ml)	2.27±1.68	2.18±1.32	0.798
AFP (ng/ml)	9.86±38.47	3.16±1.82	0.325

“-” means no value. AFP. Alpha-fetoprotein; CA19-9. Carbohydrate antigen 19-9; CA72-4. Carbohydrate antigen 72-4; CEA. Carcinoembryonic antigen; IM. Intestinal metaplasia; LGIN. Low-grade intraepithelial neoplasia; SD. Standard deviation

An independent external validation dataset was enrolled between February 2024 and April 2025, primarily from Cixian Cancer Hospital. After applying the same exclusion and frequency‑matching criteria, 149 of the 212 initially recruited participants were retained in the final analysis, comprising 79 gastric cancer patients, 40 patients with benign gastric diseases, and 30 healthy controls (Fig. 1**;** Table 2).

**Table 2:** Clinical characteristics of enrolled subjects for independent validation dataset.

**Clinical indexes**	**Gastric cancer group (*****n*****=79)**	**Non-cancer group (*****n*****=70)**	***P*****-value**
Age (years, mean±SD)	59.30±10.08	56.37±7.63	0.046
Sex [*n* (%)]			0.426
Male	47 (59.49)	47 (67.14)	
Female	32 (40.51)	23 (32.86)	
Cancer stage [*n* (%)]			-
I	17 (21.52)	-	
II	23 (29.11)	-	
III	36 (45.57)	-	
IV	3 (3.80)	-	
Benign diseases [*n* (%)]			-
Chronic gastritis	-	26 (65.00)	
Gastric polyp	-	8 (20.00)	
LGIN & IM	-	6 (15.00)	
Histological subtype^a^ [*n* (%)]			-
Tubular/Papillary	16 (20.25)	-	
Poorly cohesive/Signet-ring	22 (27.85)	-	
Mixed	34 (43.04)	-	
Wall thickening [*n* (%)]			-
<1.5 cm	40 (50.63)	-	
≥1.5 cm	39 (49.37)	-	

a Histological subtype information was unavailable for 7 cases. “-” means no value. AFP. Alpha-fetoprotein; CA19-9. Carbohydrate antigen 19-9; CA72-4. Carbohydrate antigen 72-4; CEA. Carcinoembryonic antigen; IM. Intestinal metaplasia; LGIN. Low-grade intraepithelial neoplasia. SD. Standard deviation.

The diagnosis of gastric cancer and benign gastric diseases was confirmed by gastroscopy combined with histopathological examination. All gastric cancer cases were histologically confirmed as adenocarcinomas, and none had distant metastasis at the time of diagnosis. Healthy controls were defined as individuals who had no evidence of gastric disease confirmed by gastroscopy and had no history of, or current diagnosis of, any malignant neoplasm. For patients with gastric cancer, blood samples were collected prior to the initiation of any treatment.

The study protocol was approved by the Institutional Review Boards (IRBs) of all participating institutions, with the following approval numbers: Ethics Committee of National Cancer Center/National Clinical Research Center for Cancer/Cancer Hospital, Chinese Academy of Medical Sciences and Peking Union Medical College (IRB-22/457-3659), Ethics Committee of Shandong Provincial Hospital (IRB #SWYX2022-571), and Ethics Committee of Cixian Cancer Hospital (IRB-20230012). Written informed consent was obtained from all participants prior to sample collection. Participation was entirely voluntary. All participants were informed that refusal to participate would not affect their clinical care or medical treatment.

### 2.2 Public datasets for biomarker mining and panel design

**Fig. 2:**
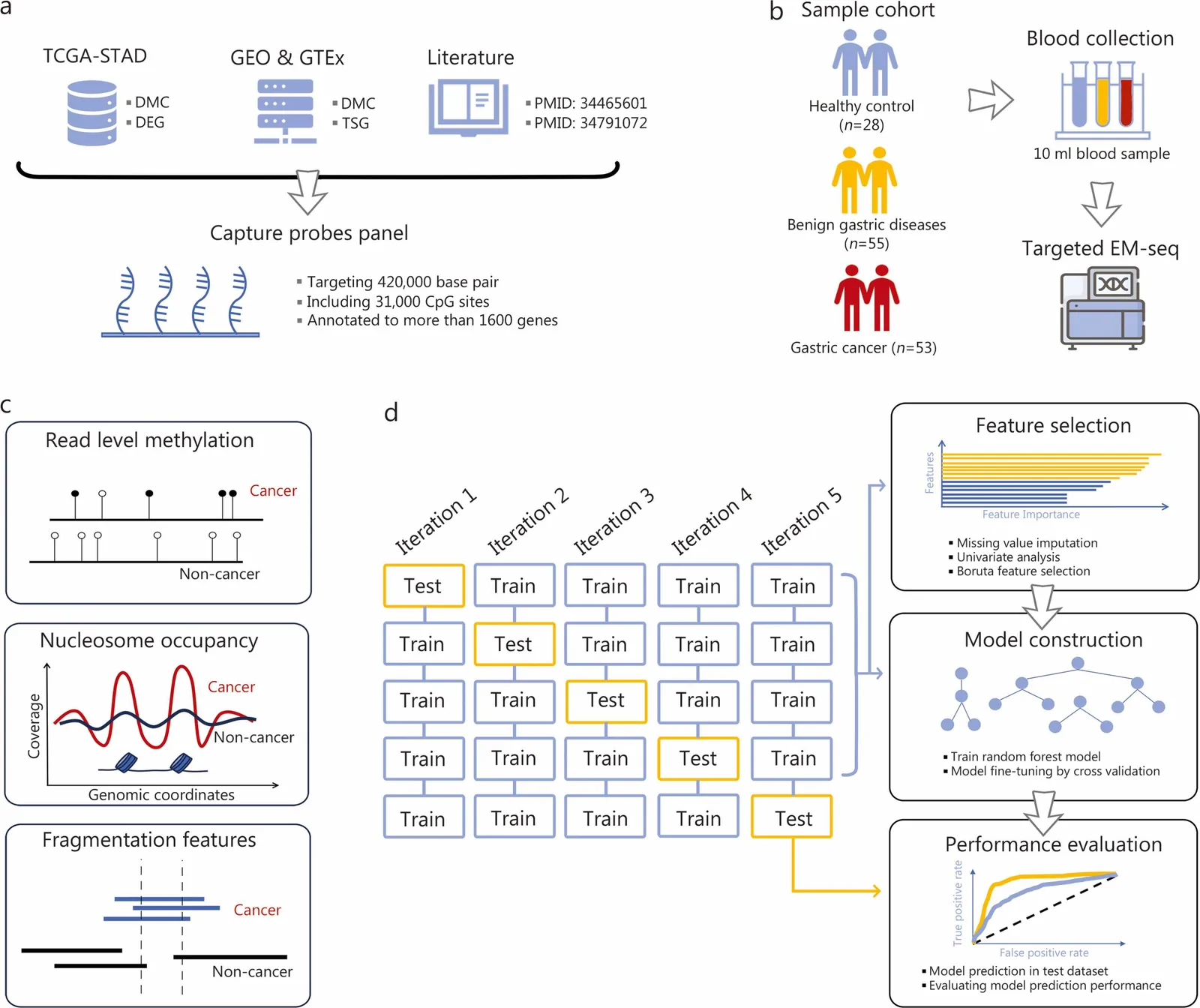
Schematic overview illustrating the development of the *GastroAlert* multimodal assay. **a** Design of the capture probe panel for the detection of gastric cancer. **b** Deep-targeted EM-seq analysis of blood samples from patients with gastric cancer, benign gastric conditions, and healthy controls. **c** Feature extraction from sequencing data across three dimensions: read-level methylation, nucleosome occupancy, and fragmentation patterns. **d** Development of the *GastroAlert* model through repeated cross-validation. DEG. Differentially expressed genes; DMC. Differentially methylated CpG sites; EM-seq. Enzymatic methylation sequencing; GEO. Gene expression omnibus; GTEx. Genotype-tissue expression; TCGA-STAD. The Cancer Genome Atlas-stomach adenocarcinoma; TSG. Tissue-specific genes.

To nominate candidate gastric cancer-associated targets for capture panel design, we leveraged publicly available tissue datasets from TCGA-STAD [13], NCBI GEO [14], and GTEx [15] (Fig. 2**a**). TCGA-STAD DNA methylation (Illumina HumanMethylation450) and RNA-seq gene expression (counts) were downloaded from the UCSC Xena Browser (https://xenabrowser.net/datapages/). An external gastric tissue methylation dataset was obtained from NCBI GEO (GSE99553). Stomach tissue-specific expressed genes were directly retrieved from the GTEx portal (https://www.gtexportal.org/home/tissue/Stomach).

Differentially methylated CpG sites were identified from TCGA-STAD 450 K and GSE99553 450 K data using the champ. DMP function in the ChAMP R package (R v2.9.10) [16] with default settings. Differentially expressed genes (DEGs) in TCGA-STAD were identified from STAR-count RNA-seq data using the DESeq2 R package (R v1.38.3) [17]. Genes with low counts were filtered out by retaining only those with counts ≥10 in at least two samples, after which differential expression analysis was performed using the DESeq function. Candidate markers for panel design were assembled from 4 sources: gastric cancer-associated differentially methylated CpG sites, differentially expressed genes, stomach tissue-specific genes, and previously reported gastric cancer biomarkers. Priority was given to markers showing consistent differential signals across datasets and/or prior biological relevance to gastric cancer. Based on this integrated marker set, a custom capture panel (*GastroAlert*) was designed to target selected methylation-associated regions and functionally relevant gastric cancer genes.

### 2.3 Deep targeted EM‑seq and feature extraction

Peripheral blood samples were collected from all participants using cfDNA collection tubes and processed to plasma following a standardized two-step centrifugation protocol. Details of sample collection, transportation, and plasma separation are provided in the **Additional file 1: Methods**. The custom *GastroAlert* capture panel was designed based on candidate biomarkers nominated from public datasets and prior reports, as shown in Fig. 2a, covering a 420 kb genomic region encompassing more than 31,000 CpG sites and 1600 genes **(Additional file 2:** Table S1**).** To generate high‑resolution epigenomic data for model development, we performed deep targeted EM‑seq on 136 plasma samples from the discovery dataset using the custom *GastroAlert* capture panel (Fig. 2**b**).

From the raw sequencing data, we extracted three core classes of epigenetic and fragmentomic features: 1) read‑level methylation patterns, 2) nucleosome occupancy profiles, and 3) DNA fragmentation characteristics (Fig. 2**c**). All three modalities were subsequently used for biomarker discovery and model construction (Fig. 2**d**).

### 2.4 Sequencing data processing

cfDNA was extracted from plasma, followed by enzymatic methylation library preparation, hybrid capture using the custom *GastroAlert* panel, and paired-end sequencing on the NovaSeq 6000 platform. The detailed protocols for cfDNA extraction, library construction, target capture, and sequencing are described in the **Additional file 1: Methods**. The raw DNA methylation sequencing data were initially evaluated for overall quality using FastQC (v0.12.1) software [18]. This step is crucial for detecting common sequencing issues such as poor base quality, overrepresented sequences, or adapter contamination. After quality assessment, TrimGalore (v0.6.10) software was employed to trim adapter sequences, remove low-quality reads, and eliminate sequences exhibiting significant M-bias, particularly at the 5’ end of reads (parameter settings: -q 20 --clip_R1 10 --clip_R2 10), which is a common artifact in bisulfite sequencing [19]. This step ensures that only high-quality, reliable data are retained for downstream analysis.

Subsequently, the processed FASTQ files were aligned to the hg38 human genome reference using BSMAP (v2.90) software with default alignment parameters [20]. To account for potential biases introduced during sequencing, the Picard MarkDuplicates (v3.2.0) tool was used to mark duplicate sequences, which may arise from PCR amplification or optical sensor artifacts [21]. The Samtools (v1.9) tool was then applied to remove any unmapped reads, non-primary alignments, and duplicates [22]. The resulting cleaned BAM files were subjected to further filtering using the alignmentSieve (Deeptools, v3.5.2) tool to exclude long DNA fragments (>200 bp) that could represent genomic DNA contamination (parameter settings: --ignoreDuplicates --maxFragmentLength 200) [23].

### 2.5 Quantification of epigenomic feature scores

Sliding windows with a 100 bp size and a 50 bp step were generated from the targeted genomic regions of the *GastroAlert* panel **(Additional file 1:** Fig. S1**)**. For each window region, overlapping aligned reads were extracted from the filtered BAM files using the Samtools (v1.22) toolkit to quantify read-level methylation score, window protection score (WPS), and nucleosome occupancy score (NOS).

For read-level methylation score quantification, we exploited a method modified from the previously reported methylation block score [24]. The definition of read-level methylation score is as follows:

$$\mathrm{Read}\;\mathrm{level}\;\mathrm{methylation}\;\mathrm{score}=\frac{1}{n}\times \sum_{i=1}^{n}\left(\frac{\sum_{j=1}^{m}l_{\mathrm{ij}}^{2}}{C_{i}}\right)$$

For a given window region, n is the total read count. On ith read, m is the number of contiguous methylation blocks, defined as runs of consecutive methylated CpG sites along the read. The term $l_{\mathrm{ij}}$ represents the number of methylated CpG sites of each block. $C_{i}$ is the total number of CpG sites on the ith read. This read-level methylation score captures the structure of methylation patterns along individual cfDNA fragments. Instead of treating each CpG independently, the score reflects both the density and the continuity of methylated CpGs along a read. Higher values indicate longer or more consistent stretches of methylated CpGs (“methylation blocks”), which are characteristic of compacted or nucleosome-protected DNA.

Nucleosome occupancy tracks were generated using the DANPOS2 (v2.2.2) tool [25]. For each nucleosome-organization target region, we summarized the signal as the mean occupancy value using the bigWigAverageOverBed utility from the UCSC tools package (v393).

To quantify the WPS for a given target region, we first defined $N_{\mathrm{total}}$ as the total number of fragments overlapping that region and defined $N_{\mathrm{span}}$ as the number of fragments that completely span the region, that is, fragments whose two ends are both located outside the region boundaries. The WPS for the target region was then calculated as the ratio $N_{\mathrm{span}}/N_{\mathrm{total}}$.

For each metric, methylation score, WPS, and NOS, we constructed a separate window-level feature matrix. Each row corresponded to a 100 bp sliding window (defined by chromosome, start, and end coordinates), and each column corresponded to one subject. The WPS and NOS matrices had an analogous structure, with the metric-specific scores for each sample stored in separate columns. Missing values in each matrix were imputed using the mean value of the corresponding sample. These window-level matrices served directly as the input for downstream feature selection and model construction.

### 2.6 GastroAlert construction

All data from the discovery dataset were analyzed using a repeated 5-fold cross-validation procedure with 10 repetitions. In each iteration, the data were partitioned into 5 equal folds, with 4 folds used for model development and the remaining fold used for validation. For each 100 bp window-level feature (methylation score, WPS, and NOS), univariate discriminatory performance was assessed directly from the raw feature values of the training set only. Specifically, we computed 1) AUC score, 2) sensitivity at a fixed specificity of approximately 0.95, and 3) the absolute mean signal difference between gastric cancer and non-cancer controls. To retain all potentially informative features while removing clearly uninformative ones, we applied loose thresholds for initial filtering: AUC ≥0.65, sensitivity ≥0.30, and an absolute signal difference of ≥0.05 for methylation score, ≥2 for WPS, and ≥10 for NOS. The AUC and high-specificity benchmarks were informed by previous DNA methylation biomarker-selection studies [26], [27]. Because methylation score, WPS, and NOS have different numerical scales, the sensitivity and modality-specific signal-difference thresholds were defined empirically as permissive screening criteria to reduce dimensionality while retaining a broad set of potentially informative features. Features meeting these criteria were passed to the Boruta function [with maxRuns=100, pValue=0.01, min.node.size=2, and num.trees=500; Boruta R package (v8.0.0)], which is suitable for biological features, for multivariate feature refinement within the training folds [28]. Random forest classifiers were then fitted using the ranger method (ranger R package v0.17.0) [29], with the hyperparameters mtry, num.trees, and sample.fraction optimized via the caret R package (v7.0-1) [30]. The held-out test fold was used exclusively for performance evaluation. Model performance was summarized as the mean AUC across all repeated 5-fold cross-validation runs. No statistical batch correction was applied in this study. To minimize technical variation, samples within each dataset were processed using the same standardized workflow for sample collection, cfDNA extraction, library construction, target capture, and sequencing.

### 2.7 Pathway enrichment analysis

To enable pathway-level interpretation of the epigenetic markers, we performed a separate feature selection step using the entire discovery dataset. The resulting set of discriminative genomic windows was then annotated to their nearest genes using the HOMER annotatePeaks.pl (v4.11) tool [31] with default parameters. This approach assigns each epigenetic feature to the closest gene based on genomic proximity. The aggregated gene list was subsequently analyzed for pathway enrichment using the Enrichr web server (https://maayanlab.cloud/Enrichr/), focusing on Molecular Signatures Database (MSigDB) hallmark gene sets [32]. To further explore potential biological differences among feature modalities, we additionally performed supplementary modality-specific enrichment analyses for markers selected from methylation, NOS, and WPS, using both MSigDB Hallmark and Gene Ontology (GO) Biological Process terms.

### 2.8 Quantification of serum protein biomarkers

To evaluate whether the cfDNA-derived epigenetic features could outperform conventional serum protein biomarkers in discriminating gastric cancer patients from non-cancer controls, we quantified 4 clinically routine serum markers as comparators: carbohydrate antigen 19-9 (CA19-9), carbohydrate antigen 72-4 (CA72-4), alpha-fetoprotein (AFP), and carcinoembryonic antigen (CEA). These markers were measured in batches to minimize inter-assay variability. Quantification was performed using a magnetic particle chemiluminescence immunoassay on the Gi2000 automated immunoassay analyzer (Beijing Strong Biotechnology, Inc., Beijing, China). Assays were conducted with the manufacturer’s reagent kits (catalog numbers: CA724050 for CA72-4, CA199050 for CA19-9, AFP100L for AFP, and CEA050 for CEA), following all recommended protocols to ensure methodological consistency and reproducibility.

### 2.9 Study outcomes

The primary outcome was discrimination of gastric cancer from non-cancer in the locked *GastroAlert* model in the independent validation set, quantified by area under the receiver operating characteristic (ROC) curve (AUC). The secondary outcome included sensitivity at a prespecified specificity of approximately 0.90 for gastric cancer detection [33], and performance (AUC) for early-stage gastric cancer (stage I–II).

### 2.10 Statistical analysis

A sensitivity and specificity of 0.85 were defined as the target performance values for distinguishing gastric cancer from non-cancer controls. At a significance level of α=0.05 and an allowable error range of 0.10, a minimum of 49 gastric cancer cases and 49 non-cancer controls were required to ensure sufficient statistical power. The discovery dataset (53 gastric cancer cases and 83 non‑cancer controls) and the independent validation dataset (79 gastric cancer cases and 70 non‑cancer controls) both met this sample size requirement.

Continuous variables are presented as mean±standard deviation (SD) and categorical variables as counts (percentages). Group differences were assessed using a two-sided Student’s *t*-test for continuous variables and a chi-square test for categorical variables. A two-sided *P*-value<0.05 was considered statistically significant.

Diagnostic performance was evaluated by AUC. Formal pairwise comparisons of AUCs were performed using the one-sided DeLong test for correlated ROC curves. A *P*-value<0.05 was considered statistically significant. The 95% confidence intervals (CIs) of AUC were estimated using nonparametric bootstrap resampling, while 95% CIs for sensitivity and specificity were calculated using the Wilson method.

## 3 Results

### 3.1 Selection and biological characterization of informative cfDNA-derived features

**Fig. 3:**
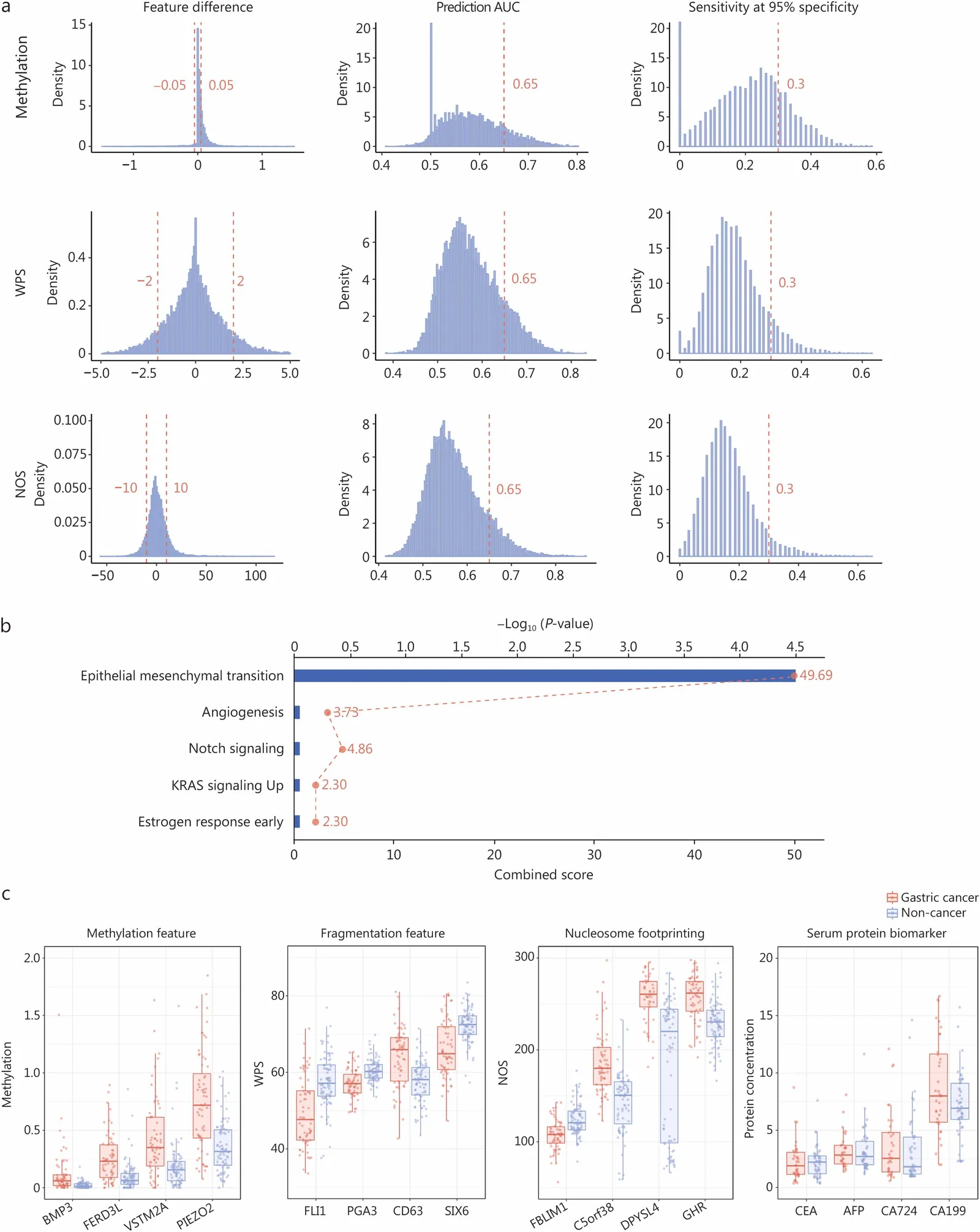
Feature selection, pathway enrichment, and superior performance of cfDNA features. **a** Histograms illustrating the univariate performance of cfDNA methylation, nucleosome footprinting, and fragmentation patterns. Feature performance is represented by the signal difference between gastric cancer and non-cancer samples, along with AUC scores and sensitivities for gastric cancer prediction. Red dashed vertical lines and red numbers indicate the preset cutoffs used for feature selection. **b** MSigDB Hallmark pathway enrichment of genes annotated from the features selected based on univariate analysis, performed using the web-based Enrichr tool. Bars represent −log10(*P*-value), and red points/labels indicate the Enrichr combined score for each pathway. **c** Boxplot depicting the top-performing cfDNA methylation markers, WPS, and nucleosome occupancy markers. Protein biomarker concentrations illustrated by boxplots. Non-cancer includes both healthy controls and benign gastric conditions. Boxes represent the interquartile range (IQR), with the lower and upper edges corresponding to the 25th and 75th percentiles, and the central line indicating the median. Whiskers extend to 1.5×IQR beyond the quartiles. AFP. Alpha-fetoprotein; AUC. Area under the receiver operating characteristic curve; CA19-9. Carbohydrate antigen 19-9; CA72-4. Carbohydrate antigen 72-4; CEA. Carcinoembryonic antigen; cfDNA. Cell-free DNA; MSigDB. Molecular signatures database; WPS. Window protection score; NOS. Nucleosome occupancy score.

Deep targeted EM-seq was completed for all 136 participants in the discovery dataset, including 53 patients with gastric cancer, 55 patients with benign gastric diseases, and 28 healthy controls **(**Table 1**)**. In contrast to traditional bisulfite sequencing, EM-seq utilizes enzymatic conversion, which minimizes cfDNA degradation, thereby preserving its integrity for comprehensive analysis [12] **(Additional file 1:** Fig. S2a**)**. Based on the customized capture probe panel, the sequencing run generated high-quality data with an average depth exceeding 500× and a conversion rate over 99.5% **(Additional file 1:** Fig. S2b**)**. Univariate evaluation showed that the discriminatory performance of most individual cfDNA features was modest, with AUC values largely clustering between 0.55 and 0.60 across methylation, WPS, and NOS features **(**Fig. 3**a; Additional file 2:** Tables S2-S4**)**. These observations indicate that markers exhibiting strong differential signals in tissue do not necessarily retain equivalent discriminatory power in plasma cfDNA, underscoring the need for subsequent feature filtering and multivariate model refinement.

Consistent with findings from public tissue-based datasets, which show that the targeted CpG sites are primarily hypermethylated in gastric cancer [34], [35], the majority of analyzed regions exhibited elevated methylation scores in plasma samples from gastric cancer patients relative to non-cancer controls (Fig. 3**a**). In contrast to the predominantly elevated methylation signals in cancer patients, the WPS and NOS features displayed a more symmetric pattern, with comparable numbers of loci showing higher or lower values in gastric cancer relative to controls **(**Fig. 3**a)**. By applying preset cutoffs, as detailed in the Method, we selected epigenetic features with significant signal differences, high AUC scores, and sensitivities for further analysis, yielding 613 methylation markers, 206 NOS markers, and 232 WPS markers (**Additional file 2:** Tables S5-S7).

Among the MSigDB hallmark gene sets, the selected epigenetic features were significantly enriched in the Epithelial-Mesenchymal Transition (EMT) pathway (Fig. 3**b; Additional file 1:** Fig. S3a), consistent with the role of EMT in gastric cancer progression [36]. To further explore the biological functions linked to each cfDNA modality, we conducted GO Biological Process enrichment analysis. Distinct enrichment patterns were observed across different modalities: methylation-related features were predominantly enriched in embryonic and tissue developmental processes; NOS features were associated with protein regulation and cell differentiation, alongside synaptic signaling; and WPS features were enriched in angiogenesis, negative regulation of cell adhesion, and cell migration (**Additional file 1:** Fig. S3b). Collectively, these findings indicate that the three cfDNA-derived modalities capture distinct yet complementary biological processes relevant to gastric cancer progression.

Notably, when comparing the top-performing cfDNA-derived epigenetic features with conventional protein biomarkers, we observed substantially better separation between gastric cancer and non-cancer controls in the epigenetic feature space **(**Fig. 3**c)**. Representative methylation markers were uniformly elevated in gastric cancer, whereas WPS and NOS markers exhibited bidirectional alterations. In contrast, the four conventional protein biomarkers showed considerably greater overlap between groups, highlighting the superior discriminatory capacity of cfDNA-based epigenetic signatures **(**Fig. 3**c)**.

### 3.2 Highly-sensitive detection of gastric cancer by the multimodal *GastroAlert* blood test

**Fig. 4:**
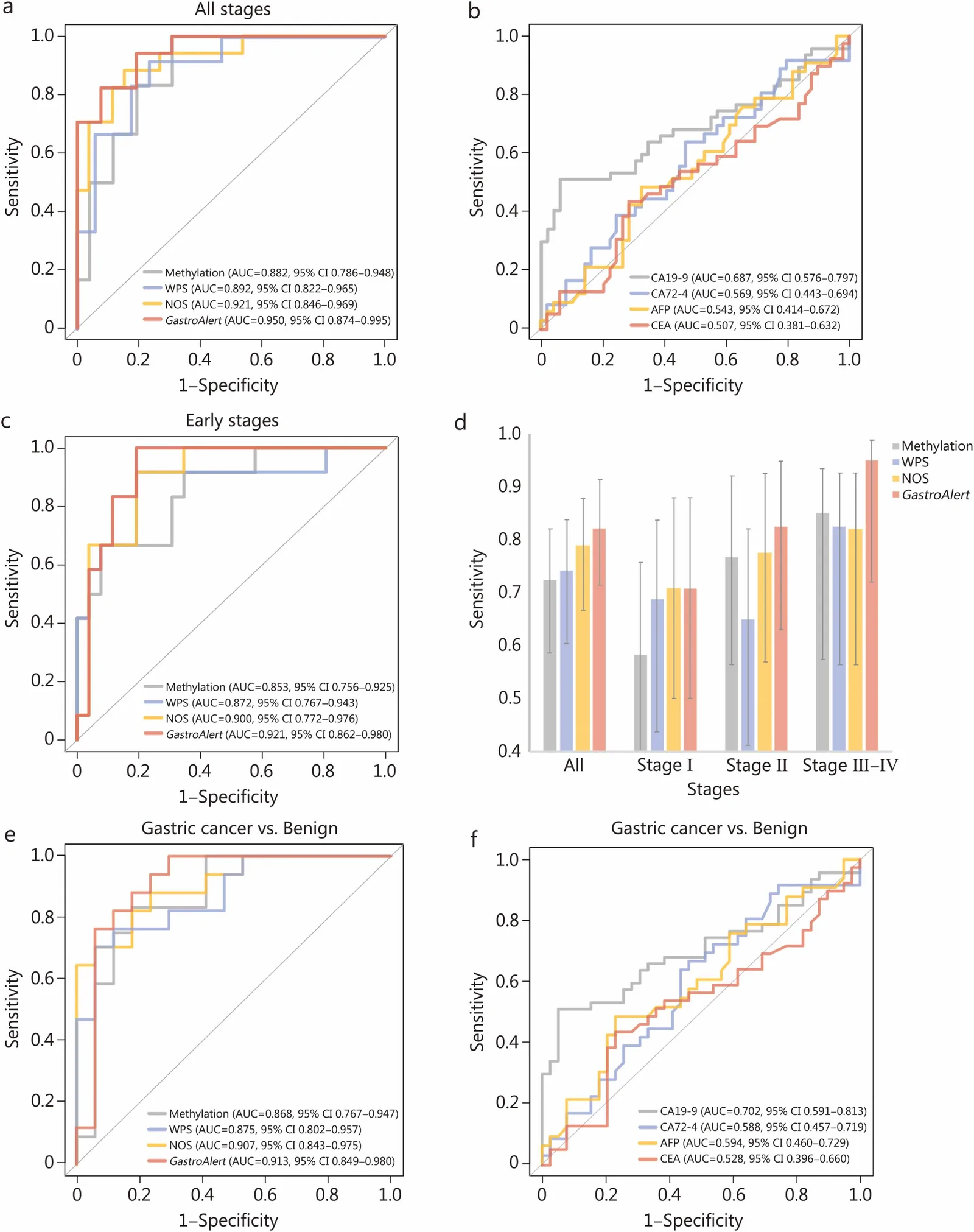
Performance of the *GastroAlert* multimodal assay in detecting gastric cancer and non-cancer controls. **a** ROC curves demonstrating the overall predictive performance of the *GastroAlert* model in comparison to unimodal models. The AUC scores represent the average of repeated 5-fold cross-validation results. **b** ROC curves depicting the prediction performance of 4 conventional protein biomarkers. **c** ROC curves illustrating the prediction performance of the models for early-stage gastric cancer in comparison to unimodal models. **d** Sensitivity of the models stratified by cancer stage. Cutoff values for each model were adjusted to maintain consistent specificity at 0.886. Error bars indicate 95% confidence intervals. **e** ROC curves in the training set comparing the multimodal *GastroAlert* model with unimodal models based on methylation, WPS, or NOS for classifying gastric cancer versus benign gastric disease samples. **f** ROC curves of 4 conventional protein biomarkers in distinguishing gastric cancer from benign gastric disease. AFP. Alpha-fetoprotein; AUC. Area under the ROC curve; CA19-9. Carbohydrate antigen 19-9; CA72-4. Carbohydrate antigen 72-4; CEA. Carcinoembryonic antigen; ROC. Receiver operating characteristic; WPS. Window protection score; NOS. Nucleosome occupancy score.

The features selected through univariate analysis were further refined and used for downstream model construction. For distinguishing gastric cancer from non-cancer controls, the multimodal *GastroAlert* model integrating methylation score, WPS, and NOS achieved an AUC of 0.950 (95% CI 0.874–0.995), compared with AUCs of 0.882 (95% CI 0.786–0.948) for methylation score only, 0.892 (95% CI 0.822–0.965) for WPS alone, and 0.921 (95% CI 0.846–0.969) for NOS alone (Fig. 4**a**). By contrast, the four conventional protein biomarkers yielded lower observed AUCs, ranging from 0.507 to 0.687 (Fig. 4**b**).

*GastroAlert* also maintained exceptional performance for the detection of early-stage (stage I–II) gastric cancer, achieving an AUC of 0.921 (95% CI 0.862–0.980), whereas the AUCs of the unimodal models did not exceed 0.900 (Fig. 4**c**). At a fixed specificity of 0.886, the *GastroAlert* model achieved a sensitivity of 0.821 (95% CI 0.708–0.908), compared with 0.724 (95% CI 0.584–0.820) for methylation score alone, 0.742 (95% CI 0.604–0.836) for WPS alone, and 0.788 (95% CI 0.665–0.880) for NOS alone (Fig. 4**d**). Stage-specific sensitivities of *GastroAlert* were 0.708 (95% CI 0.491–0.875) for stage I, 0.825 (95% CI 0.608–0.942) for stage II, and 0.950 (95% CI 0.742–0.990) for stage III–IV disease (Fig. 4**d**).

Beyond distinguishing gastric cancer from non-cancer controls, *GastroAlert* also demonstrated discriminative performance in differentiating gastric cancer from benign gastric conditions, with the highest observed AUC among the evaluated models, whereas the four conventional protein biomarkers showed lower AUC values (Fig. 4**e, f**). These findings support the potential utility of *GastroAlert* in settings where benign gastric lesions are frequently encountered, including population-level screening and clinical differential diagnosis.

### 3.3 Performance of locked *GastroAlert* model in the independent external validation dataset

**Fig. 5:**
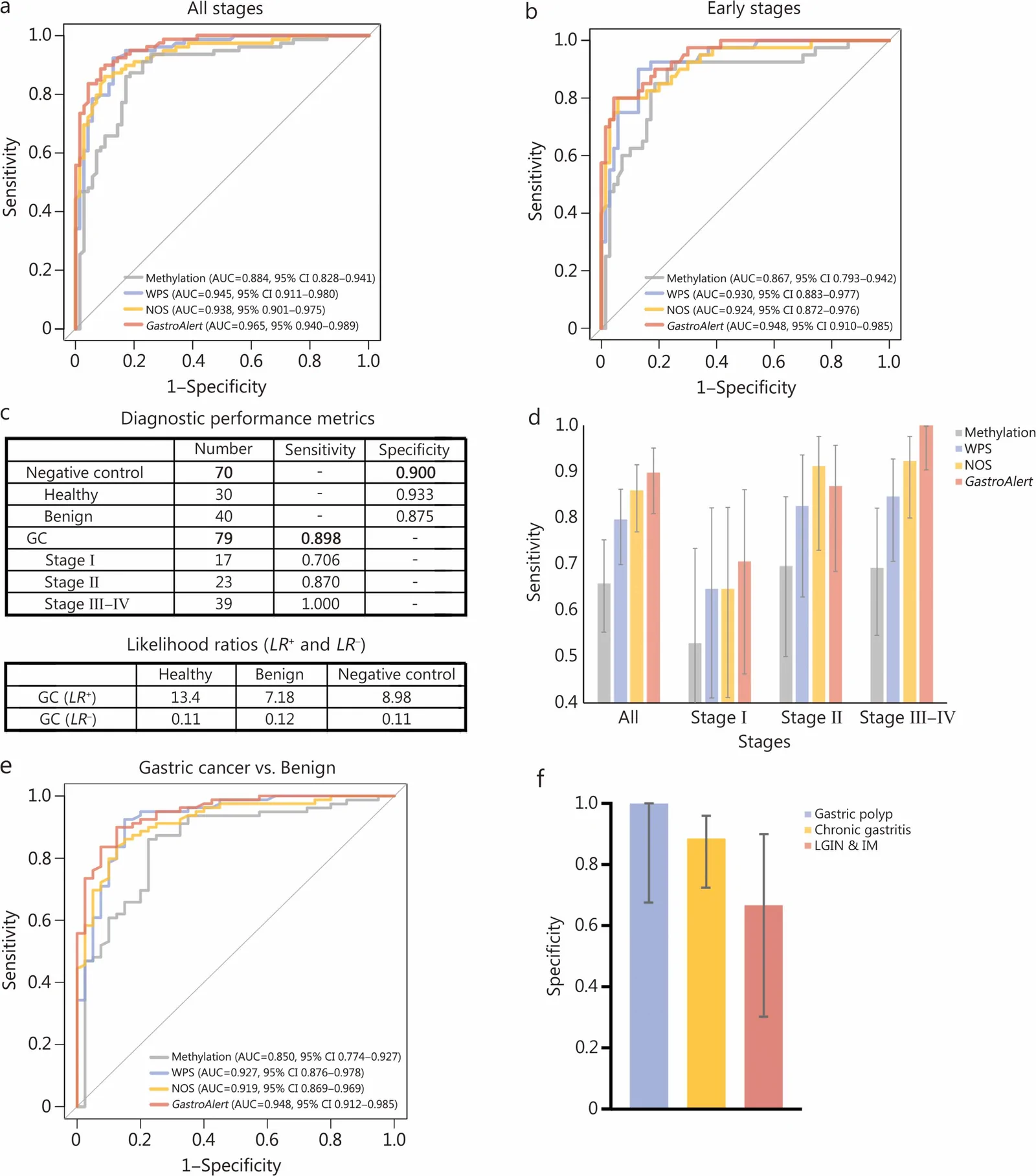
Independent validation of the *GastroAlert* multimodal assay for gastric cancer detection. **a** ROC curves showing the performance of *GastroAlert* in the independent external validation dataset for distinguishing gastric cancer from non-cancer controls, compared with unimodal models based on methylation, WPS, or NOS. **b** ROC curves evaluating model performance for early-stage gastric cancer (stage I–II) in the independent external validation dataset. **c** Diagnostic performance of gastric cancer detection across different control groups and corresponding likelihood ratios. The top table lists sample counts, sensitivity, and specificity for pairwise comparisons of gastric cancer (GC, *n*=79) against three distinct non-cancer datasets: pooled negative controls (*n*=70, healthy+benign), healthy individuals only (*n*=30), and benign gastric lesion patients only (*n*=40). Sensitivity is stratified across stage I, II, and III–IV GC subgroups. The bottom table displays matching positive (*LR*^+^) and negative likelihood ratios (*LR*^−^) for each comparison group. **d** Sensitivity of each model stratified by cancer stage in the independent external validation dataset. Error bars indicate 95% confidence intervals. Cutoffs were set to achieve the same fixed specificity of 0.900 for fair comparison across models. **e** ROC curves showing the performance of *GastroAlert* in the independent external validation dataset for distinguishing gastric cancer from benign gastric disease, compared with unimodal models based on methylation, WPS, or NOS. **f** Specificity of the *GastroAlert* model across different benign gastric disease subtypes. AUC. Area under the ROC curve; GC. Gastric cancer; IM. Intestinal metaplasia; LGIN. Low-grade intraepithelial neoplasia; ROC. Receiver operating characteristic; WPS. Window protection score; NOS. Nucleosome occupancy score.

To assess the external generalizability of the *GastroAlert* model, a critical prerequisite for clinical translation, we validated the locked (finalized) model in an independent external validation dataset consisting of 79 gastric cancer cases and 70 non-cancer controls (Table 2). For distinguishing gastric cancer patients from non-cancer controls, *GastroAlert* achieved an AUC of 0.965 (95% CI 0.940–0.989), higher than those of methylation score alone (AUC=0.884, 95% CI 0.828–0.941; *P*<0.001), WPS alone (AUC=0.945, 95% CI 0.911–0.980; *P*=0.077), and NOS alone (AUC=0.938, 95% CI 0.901–0.975; *P*=0.028) (Fig. 5**a**).

Importantly, the locked model maintained high diagnostic efficacy for early-stage gastric cancer, with an AUC of 0.948 (95% CI 0.910–0.985), compared with the unimodal counterparts (methylation score: 0.867, 95% CI 0.793–0.942; WPS: 0.930, 95% CI 0.883–0.977; NOS: 0.924, 95% CI 0.872–0.976) (Fig. 5**b**).

At a specificity of 0.900, *GastroAlert* achieved an overall sensitivity of 0.898 (95% CI 0.813–0.948) (Fig. 5**c, d**). Stage-stratified analysis confirmed robust sensitivity performance across disease stages: 0.706 for stage I (95% CI 0.469–0.867), 0.870 for stage II (95% CI 0.679–0.955), and 1.000 for stage III–IV (95% CI 0.910–1.000) (Fig. 5**c, d**). Likelihood ratio (*LR*) analysis further supported strong clinical discriminability, with a *LR*^*+*^ of 8.98 and a *LR*^*-*^ of 0.11 for distinguishing gastric cancer from non-cancer controls (Fig. 5**c**), indicating high confidence in both positive and negative test results.

We further assessed the model’s sensitivity across different histological subtypes and imaging characteristics at a specificity of 0.900. Based on the WHO histological classification, *GastroAlert* achieved a sensitivity of 0.938 (95% CI 0.717–0.989) for tubular/papillary carcinoma, 0.909 (95% CI 0.722–0.975) for poorly cohesive/signet-ring cell carcinoma, and 0.912 (95% CI 0.770–0.970) for mixed-type carcinoma (**Additional file 1:** Fig. S4a). For tumors with gastric wall thickening <1.5 cm, sensitivity was 0.786 (95% CI 0.637–0.885), compared with 0.931 (95% CI 0.807–0.978) for those with thickening ≥1.5 cm (**Additional file 1:** Fig. S4b).

We next evaluated the performance of *GastroAlert* in distinguishing gastric cancer from various benign gastric lesions. The model maintained consistent diagnostic efficacy relative to the discovery dataset (Fig. 5**e**). Subgroup analysis of benign lesions showed that *GastroAlert* achieved a specificity of 1.000 (95% CI 0.676–1.000) for gastric polyps, followed by a specificity of 0.885 (95% CI 0.710–0.960) for chronic gastritis. In contrast, the specificity for intestinal metaplasia (IM) and low-grade intraepithelial neoplasia (LGIN) decreased to 0.667 (95% CI 0.300–0.903) (Fig. 5**f**).

Collectively, these findings demonstrate the robustness, reproducibility, and external generalizability of the *GastroAlert* model for gastric cancer detection, including early-stage disease, supporting its potential clinical utility as a non-invasive diagnostic tool.

## 4 Discussion

This study demonstrates the clinical potential of *GastroAlert*, a novel multimodal epigenetic blood test that integrates DNA methylation, nucleosome footprinting, and DNA fragmentation patterns for early detection of gastric cancer. The model’s superior performance compared to conventional protein biomarkers (e.g., CA19-9) and individual epigenetic features highlights its promise as a non-invasive diagnostic tool. This advancement is particularly significant given the current limitations of existing gastric cancer screening paradigms.

Endoscopy, the current gold standard for gastric cancer detection, is effective in reducing gastric cancer incidence and mortality when implemented in screening programs [37]. However, its inherent invasiveness, associated discomfort, and relatively high cost result in poor patient compliance, limiting its widespread utility in population-wide screening [38], [39]. Protein-based serum biomarkers, while more convenient, have demonstrated suboptimal diagnostic accuracy in previous studies, with reported AUCs of 0.576 for CEA, 0.449 for AFP, 0.602 for CA72‑4, and 0.595 for CA19‑9 [40], [41], [42], findings that are largely consistent with our own observations. Such low performance substantially restricts their utility in population‑based screening settings. In contrast, this cfDNA-based multimodal model in this study demonstrates substantially improved diagnostic performance for both overall and early-stage gastric cancer detection compared with these conventional protein biomarkers.

We further demonstrated that the multimodal *GastroAlert* model outperformed all unimodal cfDNA models in the independent validation dataset, with significant improvements over the methylation‑only and NOS‑only models. Although the difference between *GastroAlert* and the WPS‑only model did not reach statistical significance, the numerical improvement (0.965 vs. 0.945) is likely attributable to the limited statistical power of the validation dataset. Of note, our sample size calculation was primarily powered to detect the prespecified performance targets for distinguishing gastric cancer from non‑cancer controls, rather than to detect small differences between competing models. This consideration should be taken into account when interpreting the pairwise comparisons among the multimodal and unimodal models.

Importantly, *GastroAlert* effectively discriminated between malignant and benign gastric conditions, including chronic gastritis, a feature particularly relevant for clinical application in high‑risk populations where benign gastric lesions are frequently encountered. These results underscore its strong potential as a non‑invasive screening tool, especially for individuals with specific occupational exposures (e.g., dust, shift work) or lifestyle‑related risk factors (e.g., irregular eating patterns, chronic psychological stress) [43]. Of note, the model showed lower specificity for premalignant lesions (IM and LGIN) compared with gastric polyps and chronic gastritis, likely reflecting shared molecular alterations between these precancerous conditions and gastric cancer. This observation warrants further investigation in larger cohorts to better define the assay's performance across the full spectrum of gastric premalignancy.

We validate the rationale for adopting the MESA-based design for *GastroAlert*, which integrates cfDNA methylation with fragmentation- and chromatin-related signals (i.e., WPS and NOS). Incorporating fragmentation patterns and nucleosome-derived features provides complementary biological information regarding cfDNA cleavage mechanisms and chromatin organization, which may reflect tumor-associated chromatin remodeling events occurring early in gastric oncogenesis. These orthogonal signals are not captured by methylation alone and likely contribute synergistically to the robust diagnostic performance observed for *GastroAlert*. Notably, the integration of these three epigenetic features was achieved using EM-seq, a technique that preserves cfDNA integrity more effectively than traditional bisulfite sequencing while enabling the detection of all three modalities in a single sequencing run [25]. Critically, this multimodal analysis does not increase sequencing costs or blood sampling requirements beyond that of unimodal methylation sequencing, with an estimated total cost of approximately 200 USD per test and a turnaround time of approximately two weeks from sample processing to final result; it only modestly augments bioinformatic workload, thus ensuring the model’s cost-effectiveness and translatability to clinical practice. Further health economic evaluations are warranted to fully assess its cost-benefit profile in population screening settings.

Although prior studies have explored multimodal cfDNA analysis for cancer detection, including a shallow whole‑methylome sequencing approach that integrated methylation, fragmentation, copy‑number alterations, and fragment‑end motifs for pan‑cancer detection at approximately 2× genome coverage [11], this study differs substantially in sequencing design, analytical resolution, and intended clinical application. While suitable for broad pan‑cancer detection, such low‑pass sequencing has limited capacity to resolve tumor‑associated epigenetic changes at specific regulatory loci with high resolution, as methylation signals are summarized over 1‑Mb regions and fragmentation features are assessed genome‑wide. This design may dilute locus‑specific signals and complicate translation to simpler PCR‑based assays. In contrast, the deep targeted strategy with an average depth exceeding 500× employed in this study enables high‑resolution profiling of methylation, WPS, and NOS across CpG‑rich and nucleosome‑scale regions, and allows for the identification of individual methylation markers that could be adapted for cost‑effective large‑scale screening. Furthermore, whereas the previous study primarily addressed pan‑cancer detection and compared cancer cases mainly with healthy controls [11], this study was specifically designed for gastric cancer detection and included patients with benign gastric conditions, a clinically relevant comparator group that is particularly important when evaluating a single‑cancer screening test for high‑risk populations.

A key finding of this study is the significant enrichment of EMT-related pathways among the discriminative epigenetic features selected for *GastroAlert*. While EMT is traditionally recognized for its role in cancer metastasis across multiple malignancies, accumulating evidence highlights its critical involvement in gastric cancer initiation, particularly in the context of *Helicobacter pylori* (*H. pylori*) infection [44], [45], a risk factor implicated in approximately 80% of gastric cancer cases in China [46]. *H. pylori* infection drives gastric epithelial EMT through the secretion of virulence factors such as CagA, which activates downstream signaling pathways (e.g., ERK/JNK, PI3K/Akt), resulting in the transcriptional repression of E-cadherin and the promotion of malignant transformation. We provide epigenetic reinforcement of this paradigm, demonstrating that molecular alterations characteristic of EMT are detectable at the epigenomic level during gastric cancer development.

Notably, the identification of EMT-related epigenetic alterations in cfDNA in this study suggests that these early oncogenic events are not confined to the primary tissue but are actively released and reflected in the bloodstream via tumor-derived cfDNA. This observation provides a plausible mechanistic foundation for the diagnostic performance of *GastroAlert*, positing that the assay captures the systemic epigenetic footprint of tumorigenic processes, including EMT. While, future studies are warranted to elucidate whether the specific epigenetic alterations we identified are directly and causally induced by *H. pylori* infection.

In addition, the analysis revealed that the nucleosome footprinting feature outperformed methylation features in distinguishing gastric cancer from non-cancer controls. This observation suggests that chromatin-level alterations, such as aberrant nucleosome positioning and occupancy, may be more prominently dysregulated in the early stages of gastric oncogenesis. These changes likely reflect underlying chromatin remodeling processes that are hallmarks of tumor initiation, such as dysregulated transcriptional control and genomic instability. The superior predictive power of nucleosome footprinting features indicates that these chromatin modifications, rather than DNA methylation alone, may serve as more reliable markers for early gastric cancer detection. Further mechanistic investigations are warranted to clarify the specific associations between nucleosome repositioning and gastric carcinogenesis, which could inform the development of more targeted epigenetic biomarkers.

Beyond gastric cancer, the multimodal analytical framework integrated with dysregulated CpG methylation, nucleosome repositioning, and aberrant cfDNA fragmentation has already demonstrated robust diagnostic efficacy in other malignancies, including colorectal cancer, hepatocellular carcinoma, and pancreatic ductal adenocarcinoma [25]. Thus, the *GastroAlert* model holds considerable promise for extending its utility to a broader spectrum of cancer types, though further validation in independent cohorts of other malignancies is warranted to substantiate this potential.

More broadly, the approach employed in this study provides a valuable paradigm for addressing the long-standing challenge of limited sensitivity inherent in single-biomarker liquid biopsy assays. This is particularly relevant in the context of multi-cancer early detection (MCED), which has emerged as a burgeoning research frontier in recent years [47], [48]. Most current MCED strategies rely on a single cfDNA feature, such as methylation profiles or fragmentomic signatures, which inherently constrains their diagnostic sensitivity [47]. In contrast, the multimodal strategy, which synergistically combines cfDNA methylation, fragmentomics, and high-resolution nucleosome footprinting, may offer a novel and more powerful framework to enhance the accuracy of MCED.

Several limitations of this study must be acknowledged. First, although the sample size provided adequate statistical power for initial model development and validation, larger multicenter studies involving diverse populations are essential to robustly confirm the generalizability and clinical utility of *GastroAlert*, particularly for the early detection of gastric cancer. Second, due to the retrospective case-control design and the well-recognized “spectrum effect” [49], diagnostic performance estimates derived from symptomatic hospital datasets may not directly translate to asymptomatic screening populations. Therefore, the utility of *GastroAlert* in asymptomatic individuals, where early detection is most impactful, remains unevaluated. Future large-scale prospective studies in asymptomatic cohorts are essential to assess its real-world performance and feasibility for gastric cancer screening. Third, the dataset lacked a sufficient number of patients with pre-cancerous lesions (e.g., high-grade intraepithelial neoplasia), which are pivotal for reducing gastric cancer incidence and mortality through early intervention. Consequently, we were unable to evaluate the diagnostic performance of *GastroAlert* for pre-cancerous lesions. Subsequent studies will focus on optimizing the model and expanding the cohort to include more patients with pre-cancerous conditions to address this gap. Finally, although the biomarker discovery process deliberately prioritized gastric cancer-specific methylation markers, we did not experimentally evaluate *GastroAlert* in other gastrointestinal malignancies (e.g., colorectal, esophageal, or pancreatic cancer). Therefore, the cancer-type specificity of the assay remains unclear. Future studies incorporating a broader range of gastrointestinal cancer controls are needed to rigorously assess its specificity for gastric cancer.

## 5 Conclusions

In summary, this study establishes the feasibility of using multimodal epigenetic markers in cfDNA for the non-invasive detection of gastric cancer. *GastroAlert* demonstrates superior diagnostic performance compared to conventional protein biomarkers and unimodal epigenetic models, particularly for early-stage gastric cancer. While these findings are promising, further multicenter, large-scale prospective studies are required to confirm the model’s diagnostic accuracy, evaluate its utility in asymptomatic populations and for pre-cancerous lesion detection, and establish its potential as a routine screening tool to improve gastric cancer outcomes.

## Abbreviations

AFP, Alpha-fetoprotein; AUC, Area under the ROC curve; CA19-9, Carbohydrate antigen 19-9; CA72-4, Carbohydrate antigen 72-4; CEA, Carcinoembryonic antigen; cfDNA, Cell-free DNA; CI, Confidence intervals; EM-seq, Enzymatic methylation sequencing; EMT, Epithelial-mesenchymal transition; IM, Intestinal metaplasia; LGIN, Low-grade intraepithelial neoplasia; MCED, Multi-cancer early detection; MESA, Multimodal epigenetic sequencing analysis; MSigDB, Molecular Signatures Database; NOS, Nucleosome occupancy score; *H. pylori*, *Helicobacter pylori*; ROC, Receiver operating characteristic; WPS, Window protection score; NGS, Next-generation sequencing;;

## Declarations

### Ethics approval and consent to participate

This study adhered strictly to the principles of the Declaration of Helsinki and the Department of Health and Human Services Belmont Report, with the approval by the ethics committee of National Cancer Center/National Clinical Research Center for Cancer/Cancer Hospital, Chinese Academy of Medical Sciences and Peking Union Medical College (IRB-22/457-3659), Shandong Provincial Hospital (IRB #SWYX2022-571), and Cixian Cancer Hospital (IRB-20230012). Written informed consent was obtained from all participants prior to sample collection. Participation was entirely voluntary. In accordance with institutional policy, participants received a transportation allowance. All participants were informed that refusal to participate would not affect their clinical care or medical treatment.

## Fundings

This work was supported by Noncommunicable Chronic Diseases-National Science and Technology Major Project (2025ZD0551700, 2025ZD0551701), CAMS Innovation Fund for Medical Sciences (CIFMS) (2025-I2M-KJ-007), and the National Natural Science Foundation of China (82273721).

## CRediT authorship contribution statement

YJX and CFX conceived and designed the study. YJX, HPC, and JZ performed initial centrifugation of blood samples to separate plasma, while JFX conducted the detection of plasma cfDNA. WQC and LS supervised and managed the project implementation. Data collection and collation were performed by JZ, CFX, JFX, DGL, HPC, and TYL. The statistical analysis and data interpretation were conducted by YJX, CFX, YJW, and JFX. The original manuscript was drafted by JFX and YJX, and revised by CFX, YJW, and WQC. All authors read and approved the final manuscript.

## Conflict of Interest

JFX, DGL, and TYL are employees of Beijing Kangshu Medical Technology Co., Ltd., which provided technical support for deep targeted enzymatic methylation sequencing. All other authors declare that they have no competing interests.

## Data Availability

The raw sequence data reported in this study were deposited at the National Omics Data Encyclopedia (www.biosino.org), under the accession numbers SUB00043018 and SUB00043050. Other data related to this study are available from the corresponding author on reasonable request.

## Footnotes

Appendix A Supplementary data associated with this article can be found in the online version at doi:10.1016/j.mmr.2026.100066.
