# Supplementary material for: GastroAlert: a multimodal epigenetic blood test for early detection of gastric cancer using cell-free DNA
Source: Mil Med Res. 2026 Aug 25;13(1):100066. doi: 10.1016/j.mmr.2026.100066 (PMC13527558; doi:10.1016/j.mmr.2026.100066)
Supplement: Supplementary file 1 [file mmc1.pdf]

Methods

Sample collection and transportation

Blood samples (10 ml) were obtained from each participant using cfDNA collection tubes (ZhiXuan Biotech, Xiamen, China) and transported to the laboratory at ambient temperature within 4 h. Upon arrival, plasma was separated from whole blood samples by a two-step centrifugation protocol: 1) 1600×*g* for 10 min (room temperature) to collect the plasma supernatant without disturbing the buffy coat, followed by 2) 16,000×*g* for 10 min at 4°C to remove residual cellular debris. Notably, this standardized sample collection and plasma separation procedure was strictly implemented for all participants, including those in both the discovery and validation datasets. The resulting plasma was aliquoted into barcoded cryovials and stored at −80°C or lower for long-term preservation.

cfDNA extraction, library preparation, target capture, and sequencing

cfDNA was extracted from 3.0 ml of plasma using the cfDNA Extraction Kit (Guangzhou Youze Biotech, Guangzhou, China). Proteinase K was added according to the manufacturer's instructions to digest plasma proteins, after which the digested samples were applied to spin columns for cfDNA purification. Purified cfDNA was recovered using elution buffer and quantified by Qubit fluorometry.

Enzymatic methylation sequencing libraries were prepared from 10 ng of cfDNA using the NEBNext Enzymatic Methyl-seq Kit together with Unique Dual Index Primers (New England Biolabs, Ipswich, MA, USA). Enzymatic conversion was used to minimize cfDNA degradation during library preparation. The converted libraries were hybridized to the custom-designed *GastroAlert* capture probe panel (Twist Bioscience, South San Francisco, CA, USA) to enrich fragments mapping to the predefined genomic regions. Following hybridization and purification, library quality and concentration were assessed using an Agilent Bioanalyzer (Agilent Technologies, Santa Clara, CA, USA). Libraries meeting the predefined quality-control criteria were sequenced on an Illumina NovaSeq 6000 platform (Illumina, San Diego, CA,

USA), generating 2× 150-bp paired-end reads. Each sample was required to generate at least 6 GB of sequencing data.

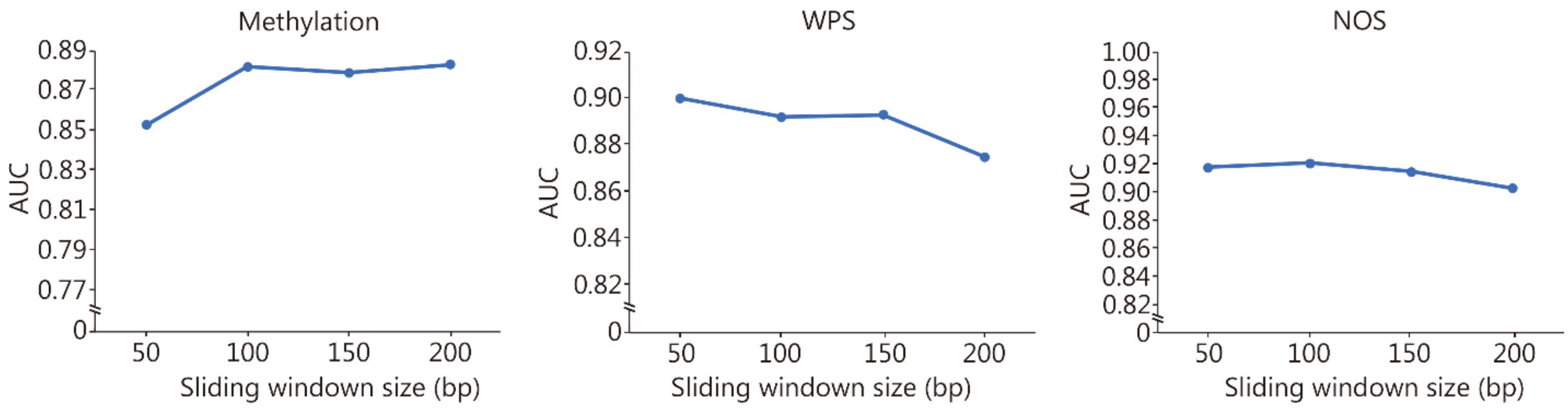

Fig. S1 Selection of sliding-window size for cfDNA feature extraction. AUC achieved by unimodal models based on cfDNA methylation, window protection score (WPS), and nucleosome occupancy score (NOS) using features extracted with different sliding-window sizes (50–200 bp). AUC. Area under the receiver operating characteristic curve; cfDNA. Cell-free DNA

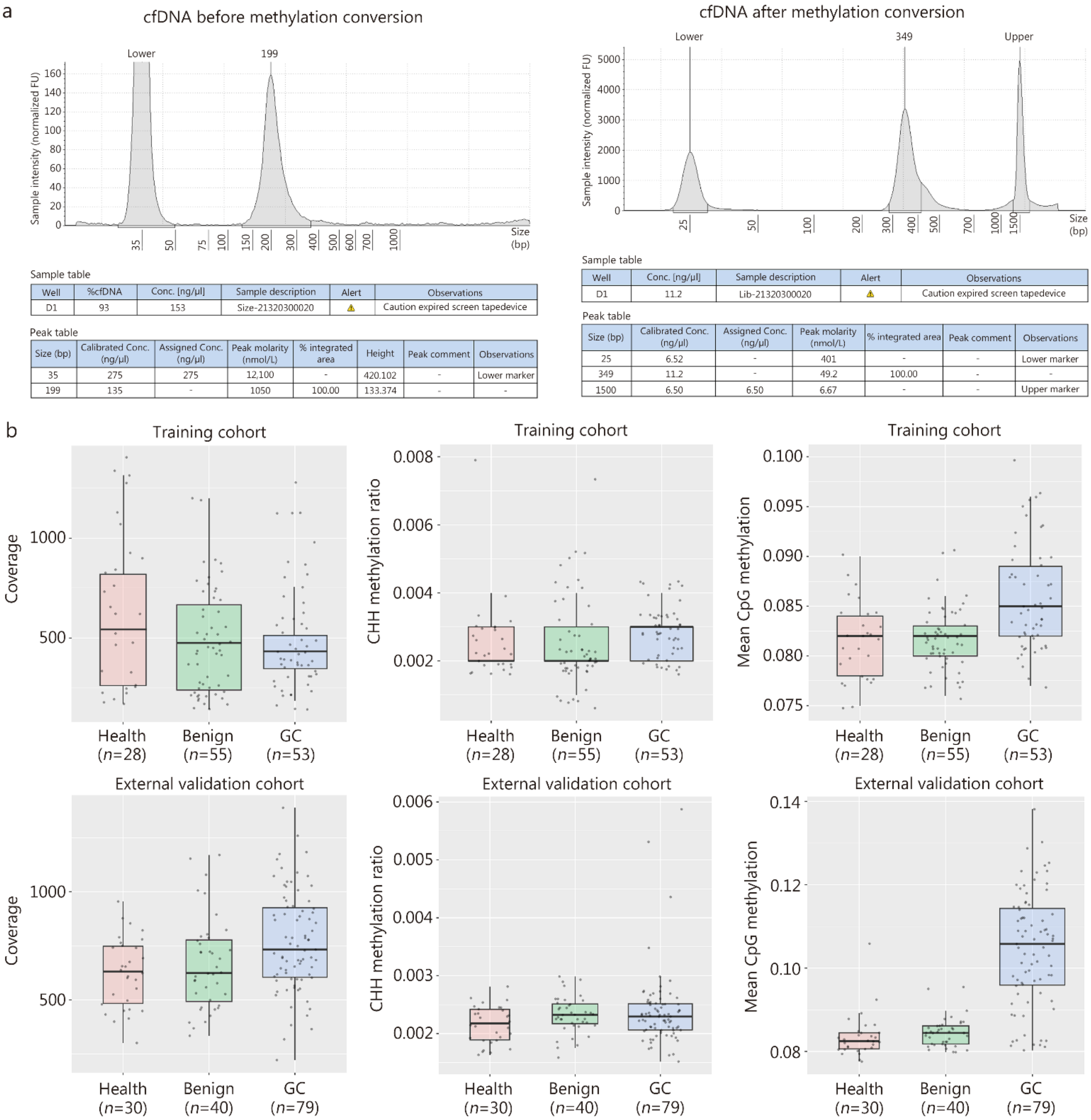

Fig. S2 cfDNA integrity and sequencing QC metrics. **a** Representative Agilent 4200 profiles showing cfDNA size distribution before and after enzymatic methylation conversion. **b** NGS quality-control metrics: on-target sequencing coverage, CHH methylation ratio, and mean CpG methylation level across targeted regions. Boxplots show the median and interquartile range (IQR) with whiskers at 1.5× IQR;

points represent individual samples. cfDNA. Cell-free DNA; Conc. Concentrations; GC. Gastric cancer; QC. Quality control

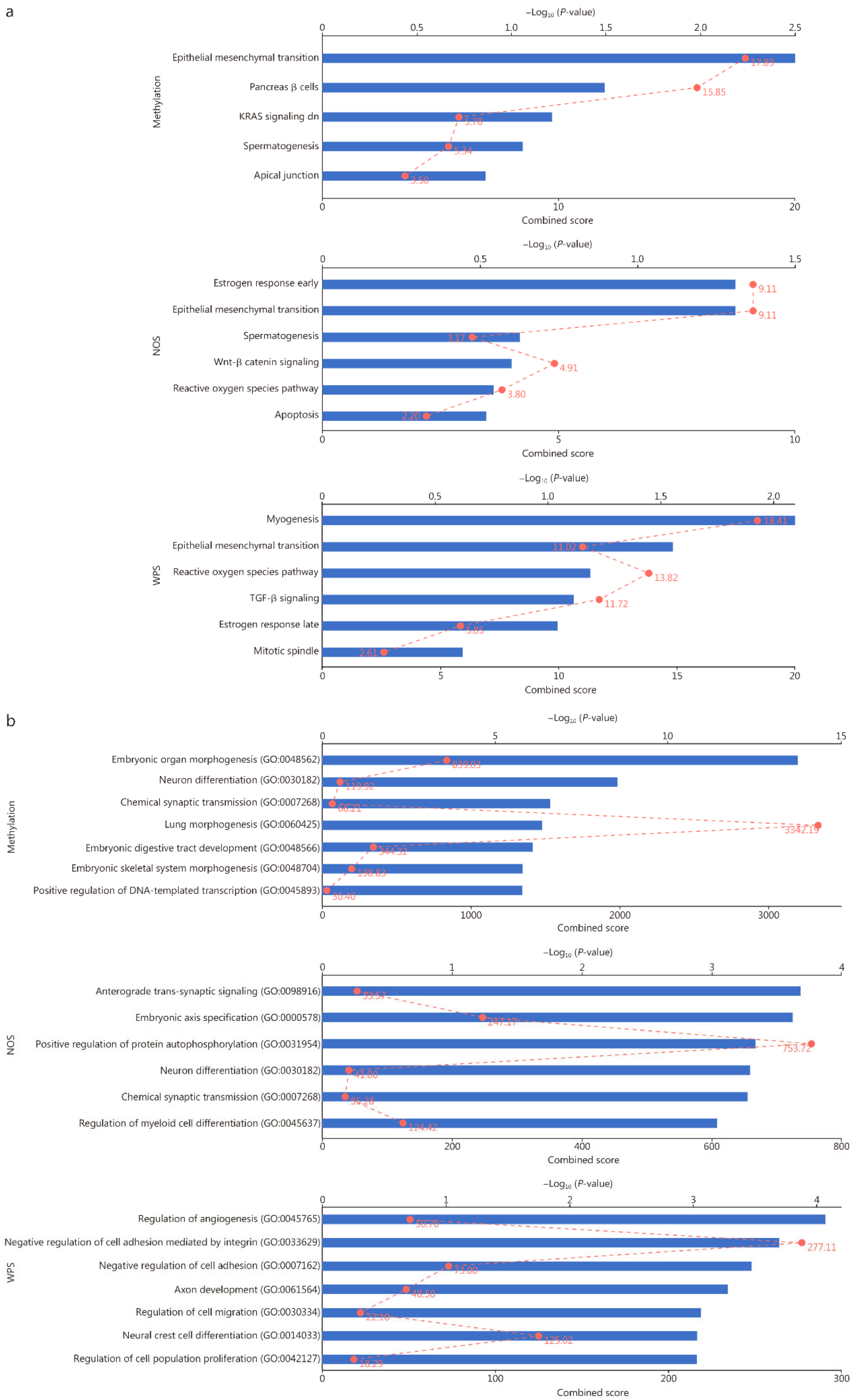

a
−Log10 (P-value)
Methylation
Epithelial mesenchymal transition
Pancreas β cells
KRAS signaling dn
Spermatogenesis
Apical junction
15.85
Combined score
−Log10 (P-value)
NOS
Estrogen response early
Epithelial mesenchymal transition
Spermatogenesis
Wnt-β catenin signaling
Reactive oxygen species pathway
Apoptosis
9.11
9.11
4.91
3.80
Combined score
−Log10 (P-value)
WPS
Myogenesis
Epithelial mesenchymal transition
Reactive oxygen species pathway
TGF-β signaling
Estrogen response late
Mitotic spindle
13.82
11.72
Combined score
b
−Log10 (P-value)
Methylation
Embryonic organ morphogenesis (GO:0048562)
Neuron differentiation (GO:0030182)
Chemical synaptic transmission (GO:0007268)
Lung morphogenesis (GO:0060425)
Embryonic digestive tract development (GO:0048566)
Embryonic skeletal system morphogenesis (GO:0048704)
Positive regulation of DNA-templated transcription (GO:0045893)
Combined score
−Log10 (P-value)
NOS
Anterograde trans-synaptic signaling (GO:0098916)
Embryonic axis specification (GO:0000578)
Positive regulation of protein autophosphorylation (GO:0031954)
Neuron differentiation (GO:0030182)
Chemical synaptic transmission (GO:0007268)
Regulation of myeloid cell differentiation (GO:0045637)
Combined score
−Log10 (P-value)
WPS
Regulation of angiogenesis (GO:0045765)
Negative regulation of cell adhesion mediated by integrin (GO:0033629)
Negative regulation of cell adhesion (GO:0007162)
Axon development (GO:0061564)
Regulation of cell migration (GO:0030334)
Neural crest cell differentiation (GO:0014033)
Regulation of cell population proliferation (GO:0042127)
277.11
Combined score

Fig. S3 Pathway enrichment analyses of selected cfDNA epigenetic features. **a** MSigDB Hallmark pathway enrichment analysis of genes annotated from the selected methylation, nucleosome occupancy score (NOS), and window protection score (WPS), performed separately using the Enrichr tool. Bars represent −log10 (*P*-value), and red points/labels indicate the combined score. **b** GO Biological Process enrichment analysis of genes annotated from the selected methylation, NOS, and WPS features, performed separately using the Enrichr tool. Bars represent $-\log_{10}$ (*P*-value), and red points/labels indicate the combined score. MSigDB. Molecular Signatures Database; GO. Gene Ontology

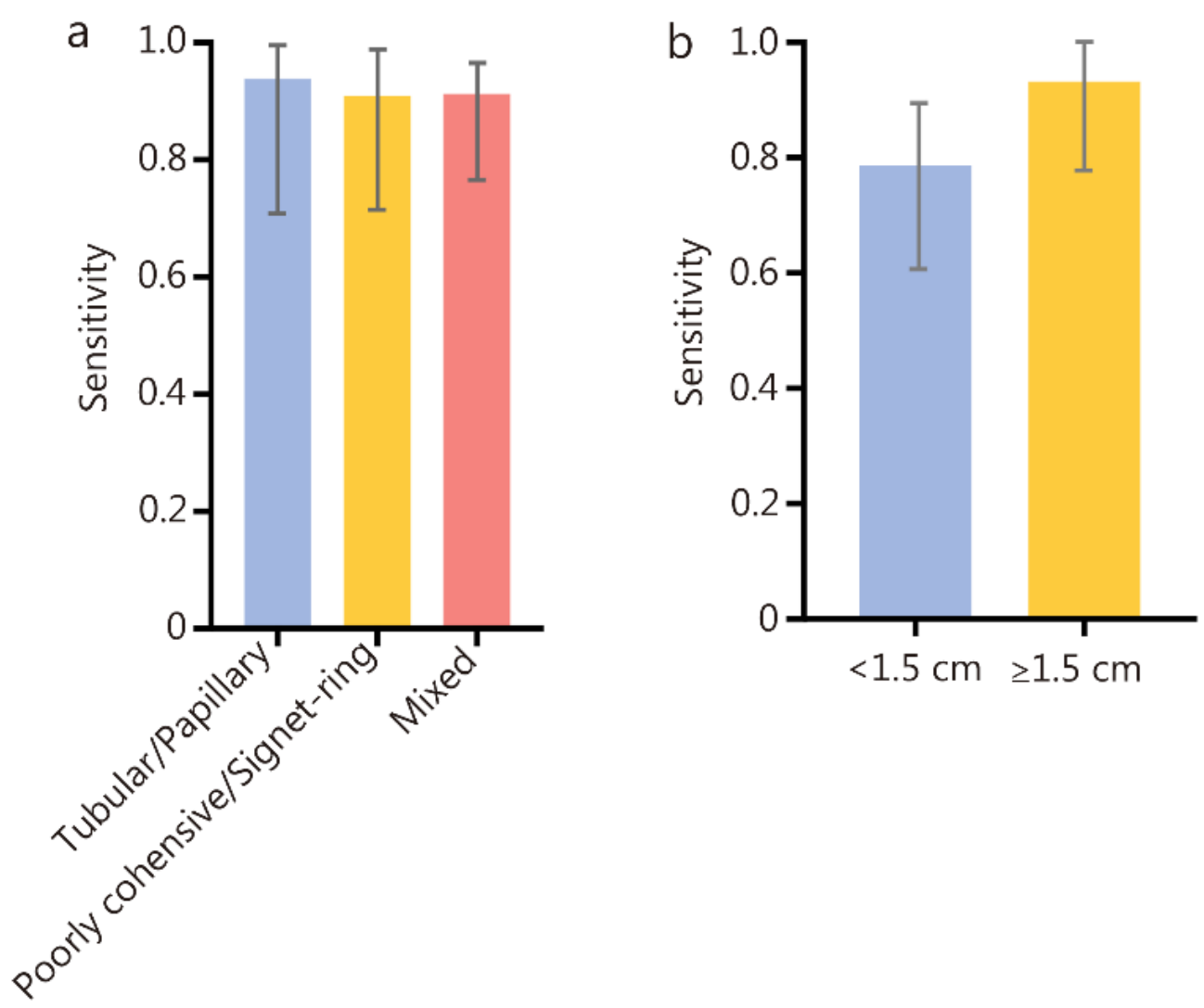

Fig. S4 Performance of the *GastroAlert* model across gastric cancer subtypes. **a** Sensitivity of the *GastroAlert* model according to WHO histological subtypes (tubular/papillary adenocarcinoma, poorly cohesive carcinoma including signet-ring cell type, and mixed subtype) at a specificity of 0.900. **b** Sensitivity of the *GastroAlert* model stratified by gastric wall thickness (<1.5 cm vs. ≥1.5 cm) at a specificity of 0.900. Error bars indicate 95% confidence intervals.
